# Hot food and beverage consumption and the risk of esophageal squamous cell carcinoma: A case-control study in a northwest area in China

**DOI:** 10.1097/MD.0000000000009325

**Published:** 2017-12-15

**Authors:** Wei-Ping Tai, Guo-Ji Nie, Meng-Jie Chen, Tajigul Yiminni Yaz, Arzi Guli, Arzigul Wuxur, Qing-Qing Huang, Zhi-Gang Lin, Jing Wu

**Affiliations:** a Department of Gastroenterology, Beijing Shijitan Hospital, Capital Medical University, Beijing; b The Digestive Endoscopy Center, Hotan District People's Hospital, Xinjiang Autonomous Region, China.

**Keywords:** case-control study, China, esophageal squamous cell carcinoma, northwest, temperature

## Abstract

**Background:**

This study was trying to investigate the association of hot food and beverage consumption and the risk of esophageal squamous cell carcinoma in Hotan, a northwest area of China with high risk of esophageal squmous cell carcinoma.

**Methods:**

A population-based case-control study was designed. For the study, 167 patients diagnosed with esophageal squamous cell carcinoma were selected from Hotan during 2014 to 2015, and 167 community-based controls were selected from the same area, matched with age and sex. Information involved of temperature of food and beverage intake was obtained by face-to-face interview. Logistic regression analyses were performed to investigate the association between temperature of food and beverage intake and the risk of esophageal squamous cell carcinoma.

**Results:**

The temperature of the food and beverage consumed by the esophageal squamous cell carcinoma patients was significantly higher than the controls. High temperature of tea, water, and food intake significantly increased the risk of esophageal squamous cell carcinoma by more than 2-fold, with adjusted odds ratio 2.23 (1.45–2.90), 2.13 (1.53–2.66), and 2.98 (1.89–4.12).

**Conclusions:**

Intake of food and beverage with high temperature was positively associated with the incidence of esophageal squamous cell carcinoma in Northwestern China.

## 1 Introduction

Esophageal cancer (EC) is still the fourth most common cancer in China in a recent report data and China experiences the highest incidence and rates of death from EC worldwide.^[1]^ In the year 2012, the EC was the fourth leading cause of cancer death in China, with estimated deaths of 210,900.^[1]^ And esophageal squamous cell carcinoma (ESCC) was the predominant pathological type.^[2]^ In addition, there was substantial geographic variation of the incidence within the country.^[3]^ Xinjiang Uyghur Autonomous Region is one of the high risk areas for ESCC in China, with an incidence nearly 5 times that of the national average rate.^[4–6]^ It is located in the northwest area of China and called “Asian Oesophageal Cancer Belt.”^[7]^ The etiological factors of EC have not been clearly elucidated and the risk factors may be various in different countries and areas. In Europe and USA, tobacco and alcohol drinking were reported to be the main causes/risk factors for EC, contributing to more than 90% of case occurrences.^[8]^ In China, the major risk factors were not very clear, but dietary factors were believed to play an important role in the occurrence of the disease.^[8]^

The positive association between high-temperature foods and beverages and risk of EC had been reported in several countries.^[9,10]^ A recent case-control study conducted in Jiangsu Province found a significantly elevated risk of EC due to drinking tea at high temperatures.^[11]^ A little different to other provinces in China, foods and beverages were more usually consumed at high temperature by Xinjiang residents. There had been reported with respect to the food and beverage temperature related to the EC in China.^[6,12]^ But there is no report about the temperature of food and beverage consumption and the risk of ESCC. Our case-control study, conducted in Hotan, investigated the effect of temperature intake of food and beverage on the risk of ESCC. The research was approved by the Ethics Committee of Hotan District People Hospital.

## 2 Materials and methods

The study was a population-based case-control study, which focused on the investigation of the food and beverage temperature and the risk of ESCC. The ESCC cases were recruited in Hotan District People's Hospital, which served the whole district and was the only hospital that could perform the EC surgery in the region. The patients eligible for the study were those who had been diagnosed in the hospital as having primary ESCC from January 2014 to December 2015, with a histological confirmation. They had lived in the area for at least 15 years. The age was from 40 to 70 years old. And they were alive for the interview. Medical records and pathology reports were reviewed to identify newly diagnosed patients (within the past 12 months). Pathological diagnoses were based on the World Health Organization Classification of Tumours of the Digestive System. Patients without histopathologically confirmed ESCC, and with memory problems were excluded. Of the totally 175 patients identified, 167 consented to participate in the study. Controls were recruited from inpatient at the same hospital from the Departments of Gastroenterology. They had also lived in Hotan for at least 15 years and were aged from 40 to 70 years. The control's exclusion criteria were previous diagnosis of malignant disease and with long-term medical diet and self-reported memory problems. All of the controls did not have any cancer and were not related to the patients with ESCC. In the end, 167 pairs of patients and controls were recruited and analyzed in the study. The controls recruited matched with cases by sex and age (±5 years). There were no significant differences in age, sex, and demographics between patients and controls. The study protocol was approved by the Human Research Ethics Committee of Hotan District Hospital. Written informed consent was obtained from all participants, who were assured of confidentiality of the information provided and their right to withdraw at any time without prejudice.

### 2.1 Data collection

Before data were collected, informed consent was obtained from each subject or the subject's guardian. Two well trained interviewers conducted a face-to-face interview to collect data from both patients and controls during the period from January 2014 to December 2015. All participants were interviewed by trained interviewers, usually with the presence of their relative helping them to recall past events. The structured questionnaire used composed of questions on demographic characteristics, anthropometry, past and family medical history, diet, and lifestyle such as cigarette smoking and alcohol consumption. Information on dietary habits was solicited, including whether participants drank tea and water and ate foods at various temperatures. The reference recall period for diet and lifestyle variables was set at 5 years before diagnosis for cases and 5 years before interview for controls.

The interviewers were trained to administer a hot beverage and food measurement protocol which used before.^[13]^ The participants were asked to prepare and drink a hot tea in the normal manner. Two cups were poured simultaneously into 2 identical cups about 250 mL, 1 for consumption and the other 1 for temperature measurement. A digital thermometer (WSS-586 thermometer, Shanghai Automatic Meter Factory, Shanghai, China) was placed in the interviewer's cup, and the temperature was immediately recorded at pouring. When the temperature reached 75°C the participant was asked to sip the tea and say whether it was how hot they usually drink. The procedure was repeated at 70°C and every 5°C until they reached their desired drinking temperature. The similar method was used to detect the food temperature. An alcohol drinker was defined as someone having drunk alcohol at least once per month.^[14]^ Someone who was a drinker but stopped more than 5 years before their diagnosis or the interview in the case of controls was defined as a former drinker. The current alcohol drinkers were defined as not having quit drinking alcohol within the 5 years before their diagnosis or their interview in the case of controls.^[15]^ Family cancer history was defined as the occurrence of cancer at any site among first-degree relatives, which referred to father, mother, and siblings.

### 2.2 Statistical analysis

Descriptive statistics were used to summarize the sample characteristics. Unconditional logistic regression analysis was performed to assess the effect of intake temperature on the EC risk. Both crude and adjusted odds ratios (ORs) and corresponding 95% confidence intervals (CIs) were computed. For each exposure variable (tea, water, and food), its intake temperature was categorized as low or mild and high. Other variables included in the logistic regression models were age (years), sex, education level (none/primary, secondary, tertiary), body mass index (5 years ago, kg/m^2^), smoking status (never, ever), alcohol drinking (never/seldom, often), family history of cancer in first-degree relatives (no, yes), and daily intakes of vegetables and fruits (g). A multivariate conditional logistic regression model was used to estimate the ORs and 95% CI, with adjustment of potential confounding factors. The significance level for all analyses was 0.05 (2-sided test).

## 3 Results

The average age of patients was 57.8 ± 5.8 years, which was 0.9 year older than the controls (Table 1). The sex and ethnic in ESCC and control group were same. No significant difference was observed in the proportions of smoking and alcohol drinking. The distribution of different body mass index in both groups was close to each other. And 18.6% (31/167) of the patients than the controls 12.5% (21/167) had a positive cancer history among first-degree relatives (*P* < .01) (Table 1). The patients had less fruit and vegetables consumption than controls.

**Table 1:**
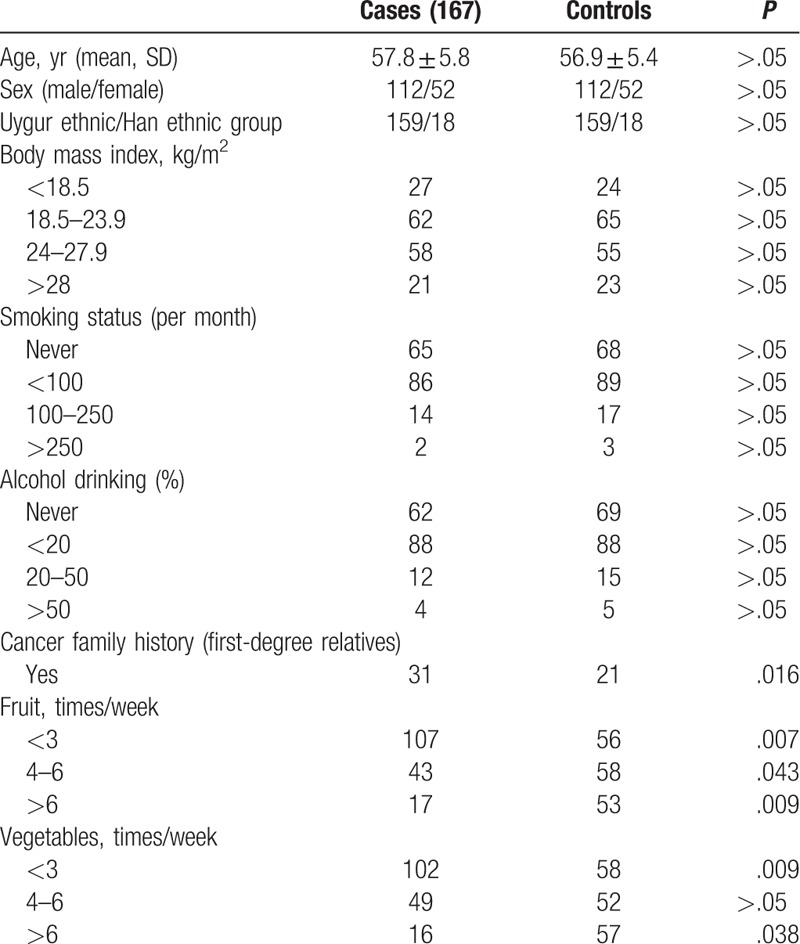
Characteristics of research subjects.

Table 2 shows the results of logistic regression for temperature of tea, water, and food intake. The ESCC patients consumed foods and beverages at higher temperatures than controls. High temperature of tea, water, and food intake significantly increase the risk of ESCC by more than 2-fold, with OR 2.15 (1.36–2.76), 2.05 (1.48–2.49), and 2.89 (1.81–4.05), with adjusted OR 2.23 (1.45–2.90), 2.13 (1.53–2.66), and 2.98 (1.89–4.12), respectively.

**Table 2:**
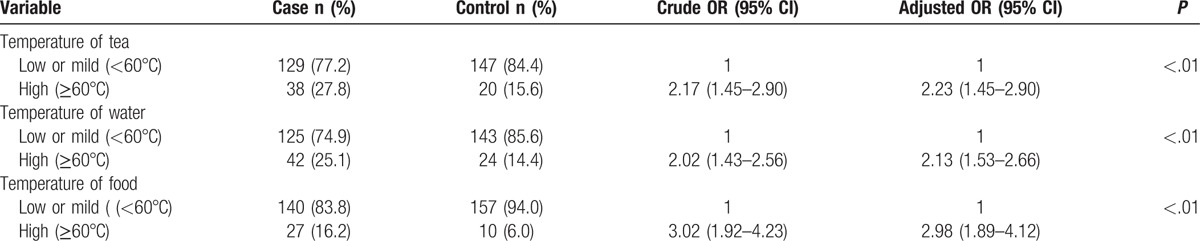
Crude and adjusted odds ratio (95% confidence interval) of esophageal squamous cell carcinoma risk in relation to temperature of food and beverage intake.

## 4 Discussion

Most EC were derived from the squamous epithelia of the esophagus and named ESCC. In recent decades, esophageal adenocarcinoma derived from islands of columnar cells near the gastroesophageal junction, has become the major type of EC in Western societies, and in China the major pathological type was still ESCC. These 2 types of EC are apparently caused by different etiological factors and we mainly discussed ESCC. The major risk factors for ESCC seemed to be tobacco smoking and alcohol drinking in some research.^[16,17]^ The ESCC risk is estimated to increase 3- to 7-fold in current smokers^[18]^ and 3- to 5-fold if due to the consumption of alcoholic beverages^[19]^; these 2 risk factors are multiplicative in smokers who are also frequent alcohol consumers. That is, these 2 factors act synergistically to induce ESCC. The etiology of ESCC is complicated. In some area, the dietary carcinogens and insufficiencies of micronutrients were found to be important risk factors than smoking and drinking. In many economically less-developed areas, such as Linxian in Northern China^[20]^ and Golestan in Northeastern Iran.^[21]^ Inhibition of the nitrosation reaction by polyphenols and ascorbic acid in fruits and vegetables was proposed as a mechanism for the protective roles of fruits and vegetables against ESCC polycyclic aromatic hydrocarbons, which are produced by the burning of wood or coal for cooking or room heating in poorly ventilated rooms and were suggested as possible etiological factors.^[20]^

Consumption of beverages and food at high temperatures, which causes thermal damage to the esophageal epithelium, has been documented to increase the risk of ESCC.^[22]^ Similarly, swallowing hard food without sufficient chewing due to dental problems may cause physical irritation to the esophageal epithelium and increase the risk of cancer. In certain areas, such as Taiwan and India, betel quid chewing, which irritates the oral and esophageal mucosa, increases the risk of ESCC, especially when used in combination with tobacco.^[23]^ This inconsistency could be due to geographical variations or due to contaminations or artifacts. A recent meta-analysis showed an association between human papillomavirus infection and ESCC in the Chinese population.^[24]^ Our study provided the first report on the positive association between high-temperature food and beverages and the risk of ESCC in northwest China, an area with high incidence of EC in China and in the world. Our findings are consistent with a previous case-control study conducted in Guangdong Provinces, in the South of China, which suggested that intake of hot beverages could significantly increase the risk of EC.^[10]^ In another case-control study from Heilongjiang Province of China, high temperature of meals and drinks was identified as a strong risk factor for EC.^[25]^ Similar results were also reported in Jiangsu Province.^[11]^ Also there is negative correlation report between hot liquid intake and EC according to a prospective cohort study conducted in Shanxi Province of China.^[26]^ A research reported the correlation between hot food and liquid intake and EC.^[6]^ In Africa, the hot beverage also proved to be contribute to the EC.^[27]^

There are some possible mechanisms through which high-temperature food and beverages contribute to the initial and development of ESCC, and involve both direct and indirect pathways. The chronic thermal irritation of esophageal mucosa might stimulate the endogenous formation of reactive nitrogen species and subsequently form carcinogenic nitrosamines. It has also been hypothesized that the repeated thermal injury may impair the barrier function of the esophageal epithelium, thereby making it more vulnerable to the damage by intraluminal carcinogens,^[22,28]^ especially in ESCC.^[29]^

Hotan Renmin Hospital is the local medical center in this region. The ESCC patients with a pathological diagnosis between 2014 and 2015 were recruited for the present study and interviewed in the hospital. Furthermore, the controls, with no diagnosed cancer history, especially for upper gastrointestinal cancers, were found by the hospital. The participation rate was >95% among patients and >95% among the controls. The patients and the controls were matched individually (1:1), to control the confounding effects of age and sex for the analyses between variables and ESCC risk. To avoid misclassification of the case-control status, we recruited only incident patients who had been diagnosed with ESCC within the past 12 months and subsequently confirmed with pathology. All controls were carefully screened. In the data analysis, conditional logistic regression was used to investigate the effects of certain factors and to control the influences of confounders.

One of the limitations in the present study was the recall bias, which might occur in any case-control study. We tried to minimize the bias: interviewers had standardized training regarding data collection and the subjects were unaware of the hypotheses of the study. The sample size was limited as well, resulting in a wide 95% CI; however, there was still significant association in the analyses.

To determine the effect of food and beverage temperature of ESCC, information of other exposures and confounding factors such as tobacco smoking, alcohol drinking, and fruit and vegetables intake, was also collected. It was possible that some ESCC cases might have modified their dietary behaviors since the onset of the disease. To avoid reverse causation, the reference period for the recall of intake habits was set at 5 years before diagnosis for cases and five years before interview for controls.

Our hospital served the entire region, the participants could be considered as representative of the target population of Hotan District. Also, there is shortcoming of our research. The most prominent, scientifically supported, and long-regarded risk factors for EC are tobacco, alcohol, and reflux esophagitis. In our research, the proportion of tobacco, alcohol in our region is small for >96% of the residents were Uygur and only a few people in this ethnic group smoke and drink alcohol. The other risk factor such as salted foods, nitrogen compounds, and carcinogens, may be overlapped with the temperature aspect.^[30]^

In conclusion, this study found that intake of food and beverage at high temperature was positively associated with the incidence of ESCC in northwest area of China. Prospective cohort studies with actual temperature measurements of foods and beverages are recommended to confirm the observed findings. In the meantime, it is advisable to avoid the intake of hot food and beverages for the prevention of ESCC in this high-risk area of China. There was report that theory of planned behavior could reduce hot tea consumption among Iranian students and prevent the EC.^[31]^ We had given some health education speech in the TV and through newspaper and the Internet to help the people in this region each year. The risk of tobacco and alcohol was not significant in our sample. It will be useful to further study the associations between the intake of food and beverage at high temperature and ESCC risk separately, with a larger sample size and a prospective study design.

## Acknowledgments

The authors are indebted to the esophageal cancer patients and control participants who agreed to be interviewed. Thanks are also due to the medical and nursing staff of the participating hospitals for their assistance in patient recruitment.
